# Relationship between anticancer sensitivities and cellular respiration properties in 5‐fluorouracil‐resistant HCT116 human colorectal cancer cells

**DOI:** 10.1002/2211-5463.13611

**Published:** 2023-04-19

**Authors:** Chinatsu Kurasaka, Nana Nishizawa, Haruka Uozumi, Yoko Ogino, Akira Sato

**Affiliations:** ^1^ Department of Biochemistry and Molecular Biology, Faculty of Pharmaceutical Sciences Tokyo University of Science 2641 Yamazaki, Noda Chiba 278‐8510 Japan; ^2^ Kowa Company Ltd. Nihonbashi‐Honcho, Chuo‐ku Tokyo 103‐8433 Japan; ^3^ Department of Gene Regulation, Faculty of Pharmaceutical Sciences Tokyo University of Science 2641 Yamazaki, Noda Chiba 278‐8510 Japan

**Keywords:** 5‐fluorouracil, cellular respiration, colorectal cancer cell, drug resistance, glucose restriction, HCT116

## Abstract

5‐Fluorouracil (5‐FU) is widely used for colorectal cancer (CRC) treatment; however, continuous treatment of CRC cells with 5‐FU can result in acquired resistance, and the underlying mechanism of 5‐FU resistance remains unclear. We previously established an acquired 5‐FU‐resistant CRC cell line, HCT116R^F10^, and examined its biological features and 5‐FU resistance mechanisms. In this study, we evaluated the 5‐FU sensitivity and cellular respiration dependency of HCT116R^F10^ cells and parental HCT116 cells under conditions of high‐ and low‐glucose concentrations. Both HCT116R^F10^ and parental HCT116 cells were more sensitive to 5‐FU under low‐glucose conditions compared with high‐glucose conditions. Interestingly, HCT116R^F10^ and parental HCT116 cells exhibited altered cellular respiration dependence for glycolysis and mitochondrial respiration under high‐ and low‐glucose conditions. Additionally, HCT116R^F10^ cells showed a markedly decreased ATP production rate compared with HCT116 cells under both high‐ and low‐glucose conditions. Importantly, glucose restriction significantly reduced the ATP production rate for both glycolysis and mitochondrial respiration in HCT116R^F10^ cells compared with HCT116 cells. The ATP production rates in HCT116R^F10^ and HCT116 cells were reduced by approximately 64% and 23%, respectively, under glucose restriction, suggesting that glucose restriction may be effective at enhancing 5‐FU chemotherapy. Overall, these findings shed light on 5‐FU resistance mechanisms, which may lead to improvements in anticancer treatment strategies.

## Graphical abstract

Glucose restriction enhanced 5‐FU chemotherapy and reduced the ATP production rate for both glycolysis and mitochondrial respiration in 5‐FU‐resistant HCT116R cells and parental HCT116 cells.

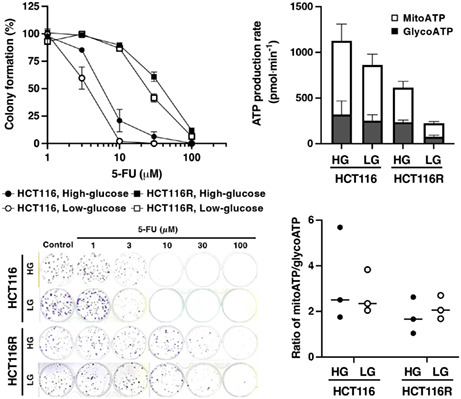

**Abbreviations**
5‐FU: 5‐fluorouracil
ECAR: extracellular acidification rate.
HG: high‐glucose
LG: low‐glucose
OCR: oxygen consumption rate

Colorectal cancer (CRC) is the third most deadly cancer in the world [1]. 5‐Fluorouracil (5‐FU) is the most important anticancer medicine for CRC treatment [2, 3]. Following administration, 5‐FU is converted to three active metabolites: fluorodeoxyuridine monophosphate (FdUMP), fluorodeoxyuridine triphosphate (FdUTP), and fluorouridine triphosphate (FUTP) [2, 3, 4, 5]. Among these, FdUMP has been found to strongly inhibit thymidylate synthase (TYMS) by forming a covalent complex with TYMS and 5,10‐methylenetetrahydrofolate [2, 3, 5]. This covalent ternary complex inhibits the TS enzyme, depletes the intracellular dTTP pool, and subsequently inhibits DNA synthesis and cell proliferation [2, 3]. FdUTP and FUTP induce cytotoxicity through their incorporation into DNA and RNA, respectively [2, 3, 4]. However, previous clinical and laboratory studies indicate that continuous treatment and exposure of CRC cells to 5‐FU can result in acquired resistance [3]. Many previous studies have described the various mechanisms of 5‐FU resistance, revealing some of the characteristics of resistant cancer cells. As a resistance mechanism, *TYMS* gene amplification, which leads to mRNA overexpression and TYMS enzyme overproduction, is known as a major mechanism of resistance to fluoropyrimidines, including 5‐FU and its derivatives [6]. Interestingly, the expression of *TYMS* undergoes translational autoregulation by interacting with the TYMS enzyme and *TYMS* mRNA [7, 8, 9, 10, 11]. This translational autoregulation of *TYMS* expression is disrupted by the TYMS ligand, resulting in TYMS translational derepression and TYMS enzyme upregulation [7, 8, 11]. However, treatments have not yet been developed to circumvent this resistance mechanism.

Abnormal metabolism is considered a hallmark of cancer cells; thus, understanding this process has become an important area of research [12, 13, 14]. Unlike normal cells, which derive most of their energy from mitochondrial oxidative phosphorylation, cancer cells are known to depend on aerobic glycolysis in the presence of abundant oxygen as their primary energy source [15, 16, 17]; this process is called the Warburg effect [15, 16, 17]. Abnormal energy metabolism is a promising target for cancer treatment [18]. Recent studies have demonstrated a relationship between the glucose concentration in the cellular environment and the effects of anticancer chemotherapeutic agents, including 5‐FU, in various cancer cells [19, 20, 21]. Previous reports have indicated that high‐glucose conditions can increase the proliferation of the human CRC cell lines, SW480, SW620, LoVo, and HCT116 [19]. In addition, high‐glucose conditions attenuate cancer growth inhibition by 5‐FU in these CRC cell lines [19]. Furthermore, in the pancreatic cancer cell lines, AsPC‐1 and Panc‐1, the anticancer effects of 5‐FU have been shown to decrease in a dose‐dependent manner at high‐glucose concentrations [20]. Interestingly, high‐glucose conditions suppress 5‐FU‐induced cell death [20]. Furthermore, high‐glucose conditions enhance cell proliferation and reduce the susceptibility of cells to chemotherapeutic drugs, including 5‐FU, in gastric cancer cells [21].

In this study, we investigated the anticancer sensitivity to 5‐FU and cellular respiration dependency of 5‐FU‐resistant HCT116R^F10^ cells and parental HCT116 cells under high (25 mm) and low (5.5 mm) glucose culture conditions. We also investigated the relationship between anticancer 5‐FU activity, cellular respiration dependency, and glucose concentration in 5‐FU‐resistant HCT116R^F10^ cells and 5‐FU‐sensitive parental HCT116 cells.

## Materials and methods

### Reagents

The anticancer drug, 5‐FU, was obtained from FUJIFILM Wako Pure Chemical (Osaka, Japan). The drug was stored as a 100‐mm stock in dimethyl sulfoxide (DMSO, Sigma‐Aldrich; Merck KGaA, Darmstadt, Germany) at −20 °C.

### Cell lines and culture conditions

The human CRC cell line, HCT116, was obtained from the American Type Culture Collection (Manassas, VA, USA). HCT116R^F10^ (5‐FU‐resistant HCT116) cells were developed according to a previously described method [22]. HCT116R^F10^ and parental HCT116 cell lines were cultured as described previously [22]. Both parental HCT116 cells and HCT116R^F10^ cells were grown in Dulbecco's modified Eagle's medium (DMEM) under high‐glucose conditions (25 mm of glucose, Cat# 043‐30085, FUJIFILM Wako Pure Chemical) or low‐glucose conditions (5.5 mm of glucose, Cat# 041‐29775, FUJIFILM Wako Pure Chemical). Both types of culture media contained 10% heat‐inactivated fetal bovine serum, 100 units·mL^−1^ penicillin, and 100 μg·mL^−1^ streptomycin.

### Exome analysis

Genomic DNA extraction was performed as described previously [22]. Genomic DNA was extracted from both types of cells using a DNeasy Tissue Kit (QIAGEN, Venlo, The Netherlands). Exome sequencing analysis of parental HCT116 and HCT116R^F10^ cells was performed by APRO Life Science Institute Inc. (Tokushima, Japan) and Macrogen Global Headquarters (Seoul, Korea).

### Colony formation assay

The colony formation assay was performed as previously described [22, 23, 24, 25]. Cells were dissociated with Accutase and then suspended in the medium. Cells were then inoculated into 6‐well plates (200 cells per well) in triplicate and incubated overnight. The cells were treated with various concentrations of the drug or with a solvent (DMSO) as a negative control. After 10 days of incubation, the cells were fixed with 4% formaldehyde solution and stained with 0.1% (w/v) crystal violet, and the number of colonies in each well was counted. The culture medium was not refreshed for 11 days.

### Cell viability WST‐8 assay

Cell proliferation WST‐8 was performed as described previously [22]. Cell viability was determined using a Cell Counting Kit‐8 (WST‐8) cell proliferation assay (Dojindo, Tokyo, Japan).

### Cellular respiration analysis

Cells were dissociated with Accutase and then suspended in high‐glucose DMEM. Cells were then seeded in an Agilent Seahorse XF24 cell culture microplate (Agilent Technologies, Santa Clara, CA, USA; 6 × 10^4^ cells per well), and the plates were incubated for 24 h. The culture medium was replaced with an analysis medium, high‐ or low‐glucose XF DMEM (Cat#103575‐100, Agilent Technologies), and the oxygen consumption rate (OCR) and extracellular acidification rate (ECAR) were analyzed using an Agilent Seahorse XFe24 analyzer (Agilent Technologies). The analysis medium contained 2 mm Seahorse XF glutamine solution (Agilent Technologies), 1 mm Seahorse XF pyruvate solution (Agilent Technologies), and 25 mm glucose in the high‐glucose medium or 5.5 mm glucose in the low‐glucose medium. The OCR and ECAR were analyzed at two time points, 1 h (short period) and 24 h (long period), after replacement with fresh high‐ and low‐glucose DMEM. Additionally, the rate of ATP production was analyzed using a Seahorse XF Real‐Time ATP Rate Assay Kit (Cat# 103592‐100, Agilent Technologies), according to the manufacturer's protocol.

### Statistical analysis

Statistical analyses were performed using the graphpad prism 9 software (GraphPad Software, Boston, MA, USA). Data were presented as the mean ± standard error. Significant differences among the groups were evaluated using Student's *t*‐test, one‐way analysis of variance (ANOVA), and two‐way ANOVA followed by Tukey's multiple comparisons test. The two‐way ANOVA was performed using a general linear model to analyze the interaction between the two factors, including ‘cell line’ and ‘glucose concentration’. The *P*‐values of < 0.05 were considered statistically significant.

## Results

### Anticancer sensitivity of parental HCT116 cells and resistant HCT116R^F10^ cells to 5 FU under high‐ and low‐glucose culture conditions

**Fig. 1:**
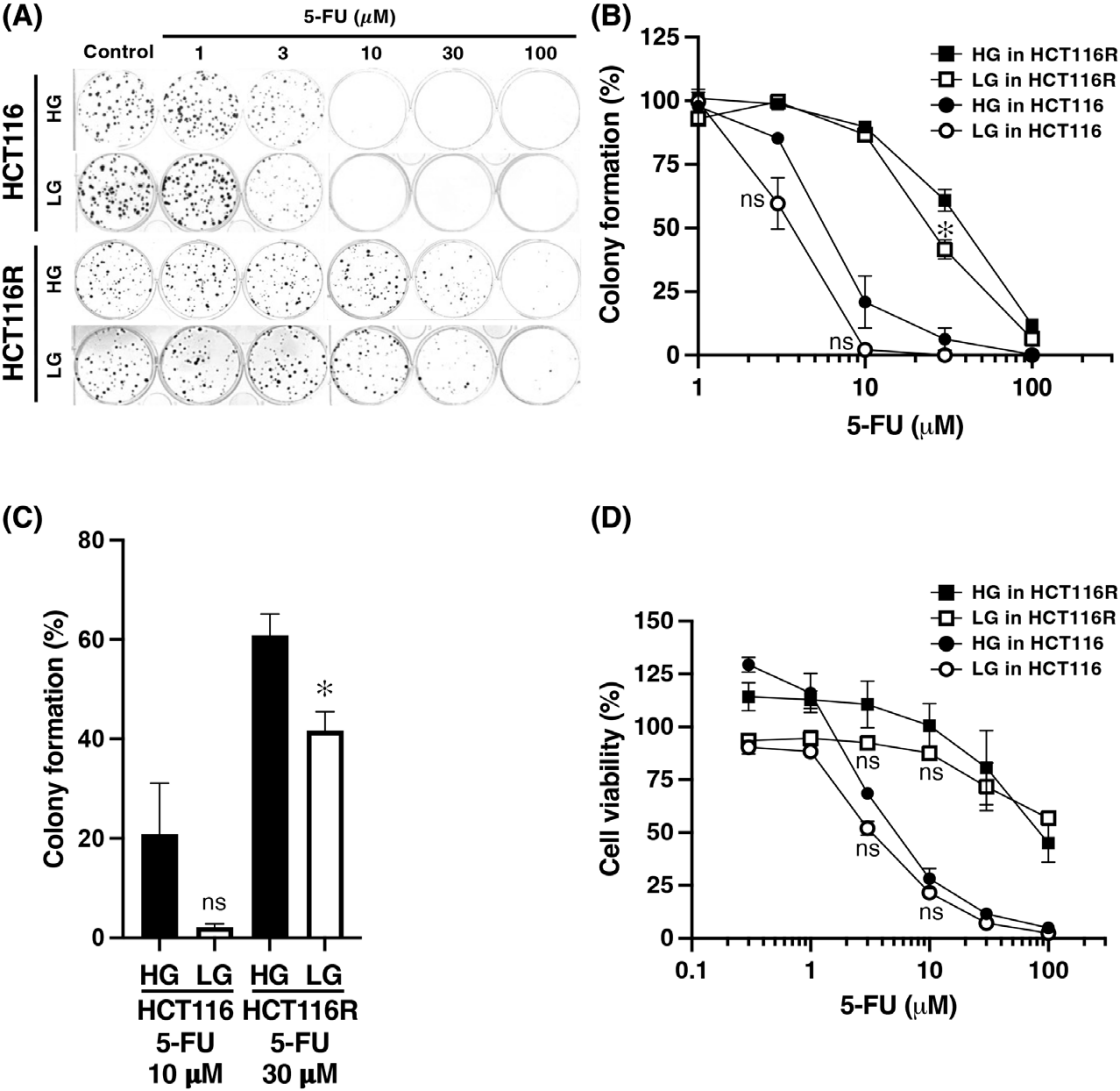
Anticancer sensitivity of 5‐FU‐resistant HCT116R^F10^ and parental HCT116 cells to 5‐FU under high‐ and low‐glucose culture conditions. (A) Anticancer sensitivities of HCT116R^F10^ and HCT116 using the colony formation assay. HCT116R^F10^ and HCT116 cells were treated with 5‐FU at concentrations of 1, 3, 10, 30, and 100 μm and subsequently incubated for 10 days. (B) Colony formation (%) represents the average of three independent experiments, with error bars showing ±SE of triplicates. Black circle, HCT116 cells under high‐glucose condition (HG in HCT116); white circle, HCT116 cells under low‐glucose condition (LG in HCT116); black square, HCT116R^F10^ cells under high‐glucose condition (HG in HCT116R); white square, HCT116R^F10^ cells under low‐glucose condition (LG in HCT116R). Student's *t*‐test, ns, not significant, *P < 0.05 (vs. HG for each cell line at same 5‐FU concentration) (C) Colony formation (%) (average of three independent experiments). Error bars show ±SE of triplicate experiments. Values were standardized by a nontreatment control in parental HCT116 and HCT116R^F10^ cells, respectively. Black bar, HG condition. White bar, LG condition. Student's *t*‐test, ns, *P < 0.05 (vs. HG for each cell line). One‐way ANOVA followed by Tukey's multiple comparisons tests, ns (HG vs. LG for each cell line). (D) The cell viability of HCT116 and HCT116R^F10^ cells was evaluated after 72 h of 5‐FU treatment with 0.3, 1, 3, 10, 30, and 100 μm. Cell viability (%) represents the average of three independent experiments, with error bars showing the ±SE of triplicates. Cell viability of 100% on the vertical axis indicates the value of the control (solvent alone) group for each condition and cell. Black circle, HG in HCT116; white circle, LG in HCT116; black square, HG in HCT116R; white square, LG in HCT116R. The one‐way ANOVA followed by Tukey's multiple comparisons test, ns (HG vs. LG for each cell line).

We investigated the resistance mechanisms of 5‐FU in HCT116R^F10^ and parental HCT116 cells, wherein we had previously revealed the genetic background through genomic analysis [22]. We examined the effect of 5‐FU on the proliferation of parental HCT116 and 5‐FU‐resistant HCT116R^F10^ cells under high and low‐glucose culture conditions using clonogenic assays. The high‐ and low‐glucose culture media contained 25 and 5.5 mm glucose, respectively. As shown in Fig. 1A,B, and Table 1, HCT116R^F10^ cells were 0.6 times (EC_50_ = 24 μm) more sensitive to 5‐FU under the low‐glucose conditions compared with the high‐glucose conditions (EC_50_ = 38 μm). Similarly, the parental HCT116 cells were 0.6 times (EC_50_ = 3.4 μm) more sensitive under the low‐glucose condition compared with the high‐glucose condition (EC_50_ = 5.5 μm; Fig. 1A,B, and Table 1). After treatment with 30 μm 5‐FU, the colony formation (%) of the HCT116R^F10^ cells was significantly decreased in the low‐glucose condition (41.7%) compared with the high‐glucose condition (60.9%; Fig. 1C). Similarly, the colony formation (%) of the parental HCT116 cells after treatment with 10 μm 5‐FU was decreased in the low‐glucose condition (2.1%) compared with the high‐glucose condition (20.9%; Fig. 1C). Furthermore, parental HCT116 cells were more resistant to 5‐FU (EC_50_ = 5.2 μm) at high‐glucose conditions than low‐glucose conditions (EC_50_ = 3.2 μm; Fig. 1D and Table 1). Alternatively, HCT116R^F10^ cells were similarly sensitive to 5‐FU in either culture condition (EC_50_ ≥ 85 μm in high glucose, EC_50_ > 100 μm in low glucose). Notably, HCT116R^F10^ cells treated with low 5‐FU concentrations of 3–10 μm had increased sensitivity to 5‐FU under low‐glucose conditions compared with high‐glucose conditions (Fig. 1D). These results suggest that glucose restriction is effective for 5‐FU sensitivity in both parental HCT116 cells and HCT116R^F10^ cells.

**Table 1:** Sensitivity of parental HCT116 and HCT116R^F10^ cells to 5‐fluorouracil (5‐FU) under high‐ and low‐glucose culture conditions. CFA, colony formation assay; EC_50_, 50% effective concentration; HG, high‐glucose condition; LG, low‐glucose condition; RI, resistance index; SI, sensitivity index; WST‐8, cell viability WST‐8 assay. RI shows the ratio of EC_50_ values between the resistant and parental cell lines; RI indicates the ratio of EC_50_ in HCT116R^F10^/EC_50_ in HCT116. SI shows the ratio of EC_50_ values between high‐ and low‐glucose conditions in parental HCT116 and resistant HCT116R^F10^ cells, respectively. SI indicates the ratio of EC_50_ in LG/EC_50_ in high‐glucose condition.

Cell line	EC_50_ (μm, CFA)			EC_50_ (μm, WST‐8)		
	HG	LG	SI	HG	LG	SI
HCT116	5.5	3.4	0.6	5.2	3.2	0.6
HCT116R^F10^	38.0	24.0	0.6	≥ 85.0	> 100.0	‐
RI	6.9	7.1	‐	≥ 16.3	> 31.3	‐

### Cellular respiration dependency of parental HCT116 cells and HCT116R^F10^ cells under high‐ and low‐glucose conditions

**Fig. 2:**
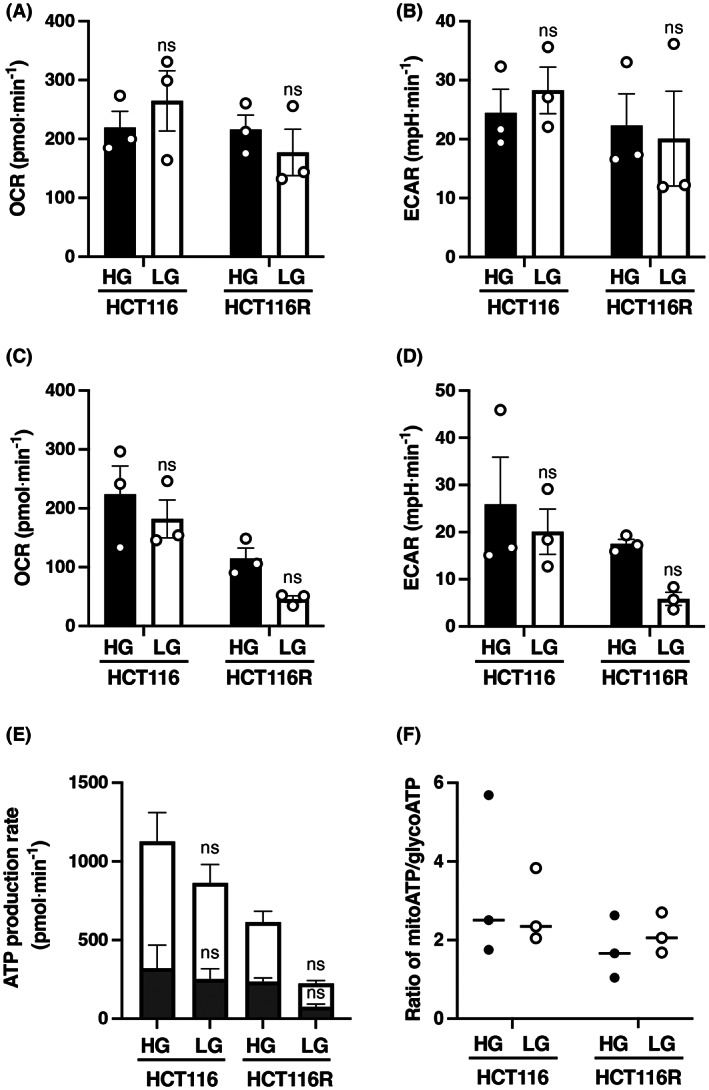
Cellular respiration dependency of 5‐FU‐resistant HCT116R^F10^ and parental HCT116 cells to 5‐FU under high‐ and low‐glucose culture conditions. (A) Oxygen consumption rate (OCR; pmol·min^−1^) at short period (1 h). Black bar, high‐glucose (HG) condition. White bar, low‐glucose (LG) condition. Two‐way ANOVA followed by Tukey's multiple comparisons test, ns, not significant (HG vs. LG for each cell line). (B) Extracellular acidification rate (ECAR; mpH·min^−1^) at a short period (1 h). The two‐way ANOVA followed by Tukey's multiple comparisons test, ns (HG vs. LG for each cell line). (C) OCR (pmol·min^−1^) at long period (24 h). Two‐way ANOVA followed by Tukey's multiple comparisons test, ns (HG vs. LG for each cell line). (D) ECAR (mpH·min^−1^) at long period (24 h). The two‐way ANOVA followed by Tukey's multiple comparisons test, ns (HG vs. LG for each cell line). OCR (pmol·min^−1^) and ECAR (mpH·min^−1^) represent the average of three independent experiments, with error bars showing ±SE. (E) Rate of ATP production (pmol·min^−1^) represents the average of three independent experiments, with error bars showing ±SE. Gray bar, glycoATP. White bar, mitoATP. The two‐way ANOVA followed by Tukey's multiple comparisons test, ns (HG vs. LG for each cell line). (F) Ratio of mitoATP to glycoATP represents average of three independent experiments. mitoATP, ATP production from mitochondria; glycoATP, ATP production from glycolysis. Solid circle, HG condition. Open circle, LG condition. HCT116, parental HCT116 cells. HCT116R, 5‐FU‐resistant HCT116R^F10^ cells.

To elucidate the association between cellular respiration dependency and 5‐FU sensitivity, we analyzed the OCR and ECAR in both the parental HCT116 cells and the 5‐FU‐resistant HCT116R^F10^ cells under high‐ and low‐glucose culture conditions using an extracellular flux analyzer. The OCR and ECAR have been identified as key indicators of mitochondrial respiration and glycolysis [26]. The OCR and ECAR of parental HCT116 and 5‐FU‐resistant HCT116R^F10^ cells were analyzed at two time points (1 and 24 h after replacement in a low‐glucose medium). As shown in Fig. 2A,B, the OCR and ECAR of the parental HCT116 cells tended to increase under the low‐glucose conditions after 1‐h treatment (OCR, 264.8 pmol·min^−1^; ECAR, 28.3 mpH·min^−1^) compared with the high‐glucose conditions (OCR, 219.4 pmol·min^−1^; ECAR, 24.5 mpH·min^−1^). Alternatively, the OCR and ECAR of the 5‐FU‐resistant HCT116R^F10^ cells tended to decrease under the low‐glucose conditions after 1 h (OCR, 177.2 pmol·min^−1^; ECAR, 20.1 mpH·min^−1^) compared with the high‐glucose conditions (OCR, 216.0 pmol·min^−1^; ECAR, 22.3 mpH·min^−1^; Fig. 2A,B). In addition, the OCR/ECAR ratio was found to be slightly higher in HCT116R^F10^ cells compared with the parental HCT116 cells under high‐glucose conditions. In contrast, the OCR/ECAR ratio was slightly lower in the HCT116R^F10^ cells compared with parental HCT116 cells under low‐glucose culture conditions. Interestingly, the OCR/ECAR ratio in the parental HCT116 cells was slightly higher under the low‐glucose conditions (ratio = 9.4) compared with the high‐glucose conditions (ratio = 9.0). Meanwhile, the OCR/ECAR ratio in HCT116R^F10^ cells was lower in the low‐glucose conditions (ratio = 8.8) compared with the high‐glucose conditions (ratio = 9.7). These data suggest that HCT116R^F10^ cells are more dependent on mitochondrial respiration in high‐glucose conditions and on glycolysis under low‐glucose culture conditions compared with the parental cells. Furthermore, parental HCT116 cells exhibited a higher cellular respiratory activity of glycolysis and mitochondrial respiration during low‐glucose culture conditions compared with high‐glucose culture conditions after 1 h.

We also analyzed the OCR, ECAR, and rate of ATP production in both cell types under high‐ or low‐glucose culture conditions after 24‐h treatment. The OCR and ECAR of the parental HCT116 cells decreased slightly under the low‐glucose conditions (OCR, 182.1 pmol·min^−1^; ECAR, 20.1 mpH·min^−1^) compared with continuous high‐glucose conditions (OCR, 223.8 pmol·min^−1^; ECAR, 25.9 mpH·min^−1^) at 24 h (Fig. 2C,D). Additionally, the OCR and ECAR of the 5‐FU‐resistant HCT116R^F10^ cells decreased under low‐glucose conditions (OCR, 45.9 pmol·min^−1^; ECAR, 5.9 mpH·min^−1^) compared with high‐glucose conditions (OCR, 115.0 pmol·min^−1^; ECAR, 17.5 mpH·min^−1^) after 24 h (Fig. 2C,D). Notably, the OCR/ECAR ratio in parental HCT116 cells was lower under the low‐glucose condition (ratio = 9.1) compared with the high‐glucose condition (ratio = 8.6). Conversely, the OCR/ECAR ratio in HCT116R^F10^ cells was higher under the low‐glucose conditions (ratio = 7.8) compared with the high‐glucose condition (ratio = 6.6).

The decreased rates of ATP production in the parental HCT116 and 5‐FU‐resistant HCT116R^F10^ cells were 23.4% and 63.5%, respectively, which decreased due to glucose restriction after the change to a low‐glucose condition (HCT116, 862.9 pmol·min^−1^; HCT116R^F10^, 224.4 pmol·min^−1^) from a high‐glucose condition (HCT116, 1126.9 pmol·min^−1^; HCT116R^F10^, 614.8 pmol·min^−1^; Fig. 2E and Fig. S1). Importantly, the ATP production rates of the HCT116R^F10^ cells were 45.4% and 74% lower under high‐ and low‐glucose conditions, respectively, compared with those of HCT116 cells. Notably, the dependence of ATP production in 5‐FU‐resistant HCT116R^F10^ cells was 61.7% for mitochondrial respiration (379.3 pmol·min^−1^) and 38.3% for glycolysis (235.5 pmol·min^−1^) under high‐glucose conditions, and 66.4% for mitochondrial respiration (149.1 pmol·min^−1^) and 33.6% for glycolysis (75.3 pmol·min^−1^) under low‐glucose conditions (Fig. 2E,F). In contrast, the dependence of ATP production in the parental HCT116 cells was 71.5% for mitochondrial respiration (805.9 pmol·min^−1^) and 28.5% for glycolysis (321.0 pmol·min^−1^) under high‐glucose conditions, and 70.8% for mitochondrial respiration (611.1 pmol·min^−1^) and 29.2% for glycolysis (251.8 pmol·min^−1^) under low‐glucose conditions (Fig. 2E,F). Notably, the glucose restriction reduced the ATP production rate for both glycolysis and mitochondrial respiration in HCT116R^F10^ cells compared with HCT116 cells. In addition, these results indicated that 5‐FU‐resistant HCT116R^F10^ cells suppress ATP synthesis from glycolysis and mitochondrial respiration more readily than parental HCT116 cells under high‐ and low‐glucose culture conditions (Fig. 2F).

In a previous report, we revealed the status of gene mutations in parental HCT116 cells and 5‐FU‐resistant HCT116R^F10^ cells through whole‐exome sequencing [22]. The gene status of several glucose metabolism pathways (e.g., glycolysis, citric acid cycle, oxidative phosphorylation)‐related genes, for example, *PGAM1*, *PKM*, *LDHB*, *NDUFS5*, *NDUFB3*, *SDHC*, *COX7A2*, *COX15*, *ATP5J2‐PTCD1*, *ATP6V1A*, *ATP6V1C1*, *ATP6V0A4*, *ATP6V0E2*, and *ATP8A1*, differs between parental HCT116 and 5‐FU‐resistant HCT116R^F10^ cells (Table 2 and Table S1). These gene mutations may contribute to the metabolic properties and 5‐FU sensitivity of parental HCT116 and 5‐FU‐resistant HCT116R^F10^ cells.

**Table 2:** Gene mutation status of glucose metabolism‐related genes in parental HCT116 and 5‐FU‐resistant HCT116R^F10^ cells. DGV, downstream gene variant; FV, frameshift variant; IV, intron variant; M, homologs mutation; MV, missense variant; SRV, splice region variant; UGV, upstream gene variant; W, wild.

Gene	HCT116	HCT116R^F10^	Effect
*PGAM1*	W	M(−1408_−1402delAAAAAAA)	UGV
*PKM*	W	M(92 + 2516_92 + 2519delAAAA)	IV
*LDHB*	M(596‐9_596‐7delTTT)	W	SRV/IV
*NDUFS5*	M(216 + 69delT)	W	IV
*NDUFB3*	M(*154_*155delAA)	W	DGV
*SDHC*	M(210‐6delT) W W	W M(77 + 43delT) M(405 + 103_405 + 104delTT)	SRV/IV VI IV
*COX7A2*	M(205‐11_205‐9delTTT)	W	IV
*COX15*	M(272 + 87_272 + 93delTTTTTTT) W	W M(272 + 88_272 + 93delTTTTTT)	IV IV
*ATP5J2‐PTCD1*	W	M(122‐7390_122‐7388delTTT)	IV
*ATP6V1A*	W	M(1762‐69_1762‐68delAA)	IV
*ATP6V1C1*	M(133‐43delT) W	W M(642‐8_642‐7delTT)	IV SRV/IV
*ATP6V0A4*	M(640‐21delT)	W	IV
*ATP6V0E2*	W	M(368 + 94_368 + 95insGTGGTTAGAGTTCTTGTTGGGATTCAGGCATCTATTTATTTCATGAAAAGAAAAGGTGGGGAGGGGAC)	IV
*ATP8A1*	W	M(1413 + 122_1413 + 125dupTTTA)	IV

## Discussion

Cancer cells usually exhibit aberrant metabolism resulting from metabolic reprogramming [15, 16, 17, 27]. This results in aerobic glycolysis as a priority over mitochondrial oxidative phosphorylation, which, in turn, provides continuous energy and nutrients to support uncontrolled proliferation. This reprogramming is known as the Warburg effect [15, 16, 17]. Increasing evidence has indicated that the glucose concentration modulates 5‐FU sensitivity in various cancer cell lines [19, 20, 21]. However, the relationship between 5‐FU resistance and respiratory dependence under glucose concentrations in CRC cells remains unclear. Our results revealed that glucose restriction enhances the anticancer effects of 5‐FU in parental HCT116 and 5‐FU‐resistant HCT116R^F10^ cells. In particular, our experiments showed that the respiration dependency of glycolysis and mitochondrial respiration on glucose restriction at short (1 h) and long (24 h) periods differs between 5‐FU‐sensitive and 5‐FU‐resistant cells. Glucose restriction at a short period in parental HCT116 cells resulted in enhanced metabolic activity for both glycolysis and mitochondrial respiration. In contrast, glucose restriction for long periods in parental HCT116 cells or at short and long periods in 5‐FU‐resistant HCT116R^F10^ cells resulted in reduced metabolic activity for both glycolysis and mitochondrial respiration. In addition, our results indicated that parental HCT116 and 5‐FU‐resistant HCT116R^F10^ cells remain dependent on mitochondrial respiration during 24‐h glucose restriction. Moreover, the ATP production rate in the long period of glucose restriction was more decreased in the 5‐FU‐resistant HCT116R^F10^ cells than in the parental HCT116 cells, indicating that 5‐FU‐resistant HCT116R^F10^ cells suppress ATP synthesis from glycolysis and mitochondrial respiration more effectively than parental HCT116 cells under both high‐ and low‐glucose culture conditions. Importantly, cellular respiration dependence under high‐ and low‐glucose conditions was reversed in both the sensitive parental HCT116 and the 5‐FU‐resistant HCT116R^F10^ cells. We considered that the metabolic properties of these resistance cells contribute to 5‐FU resistance. Previous studies have suggested that fasting exerts extensive anticancer effects in various cancers, including CRC [14, 28]. Weng *et al*. [14] reported that fasting negatively regulates glucose metabolism and proliferation in CRC via the upregulation of cholesterogenic FDFT1 mediated the suppression of AKT–mTOR–HIF1 signaling. In addition, the authors indicated in clinical significance that patients with high expression levels of *FDFT1* and low expression levels of *AKT1*, *mTOR*, *HIF1α*, *GLUT1*, and *HK2* exhibited longer survival than those with low expression levels of *FDFT1* and high expression levels of the *AKT1*‐*mTOR*‐*HIF1α* pathway and glycolytic genes [14]. Our findings further indicate that glucose restriction is effective in sensitizing 5‐FU‐resistant CRC cells to chemotherapy. Interestingly, we demonstrated that several cluster genes related to glucose metabolism (including glycolysis, the citric acid cycle, and oxidative phosphorylation) were differentially altered in HCT116 and HCT116R^F10^ cells. We further investigated the associations between cellular respiratory dependence, mitochondrial function, glucose metabolism‐related genes, and 5‐FU resistance mechanisms. Collectively, our findings provide a better understanding of 5‐FU sensitivity and resistance mechanisms and may lead to strategies to circumvent resistance to 5‐FU and its derivatives.

## Conclusion

We demonstrated that glucose restriction enhances the sensitivity to 5‐FU in both 5‐FU‐resistant HCT116R^F10^ cells and parental HCT116 cells. In addition, we revealed that 5‐FU‐resistant HCT116R^F10^ cells suppress the ATP synthesis rate of glycolysis and mitochondrial respiration more effectively than parental HCT116 cells under both high‐glucose culture conditions and low‐glucose culture conditions. Our findings may lead to improvements in anticancer treatment strategies for 5‐FU‐resistant CRC.

## Conflict of interest

The authors declare no conflict of interest.

## Author contributions

AS conceived and designed the project; CK, NN, HU, and AS acquired the data; CK, NN, HU, YO, and AS analyzed and interpreted the data, and AS wrote the paper. All the authors have read and approved the final manuscript.

## Supporting information


**Fig. S1.** Cellular respiration property of 5‐FU‐resistant HCT116R^F10^ and parental HCT116 cells to 5‐FU under high‐ and low‐glucose culture conditions.

**Table S1.** Gene mutation status of glucose metabolism‐related genes in the parental HCT116 cells and 5‐FU‐resistant HCT116R^F10^ cells.

## Data Availability

The data generated in the present study may be requested from the corresponding author.
