# Supplementary material for: Relationship between anticancer sensitivities and cellular respiration properties in 5‐fluorouracil‐resistant HCT116 human colorectal cancer cells
Source: FEBS Open Bio. 2023 Apr 19;13(6):1125–33. doi: 10.1002/2211-5463.13611 (PMC10240340; doi:10.1002/2211-5463.13611)
Supplement: Supplementary file 1 — Fig. S1. Cellular respiration property of 5‐FU‐resistant HCT116RF10 and parental HCT116 cells to 5‐FU under high‐ and low‐glucose culture conditions. [file FEB4-13-1125-s002.pdf]

Supplementary Figure 1

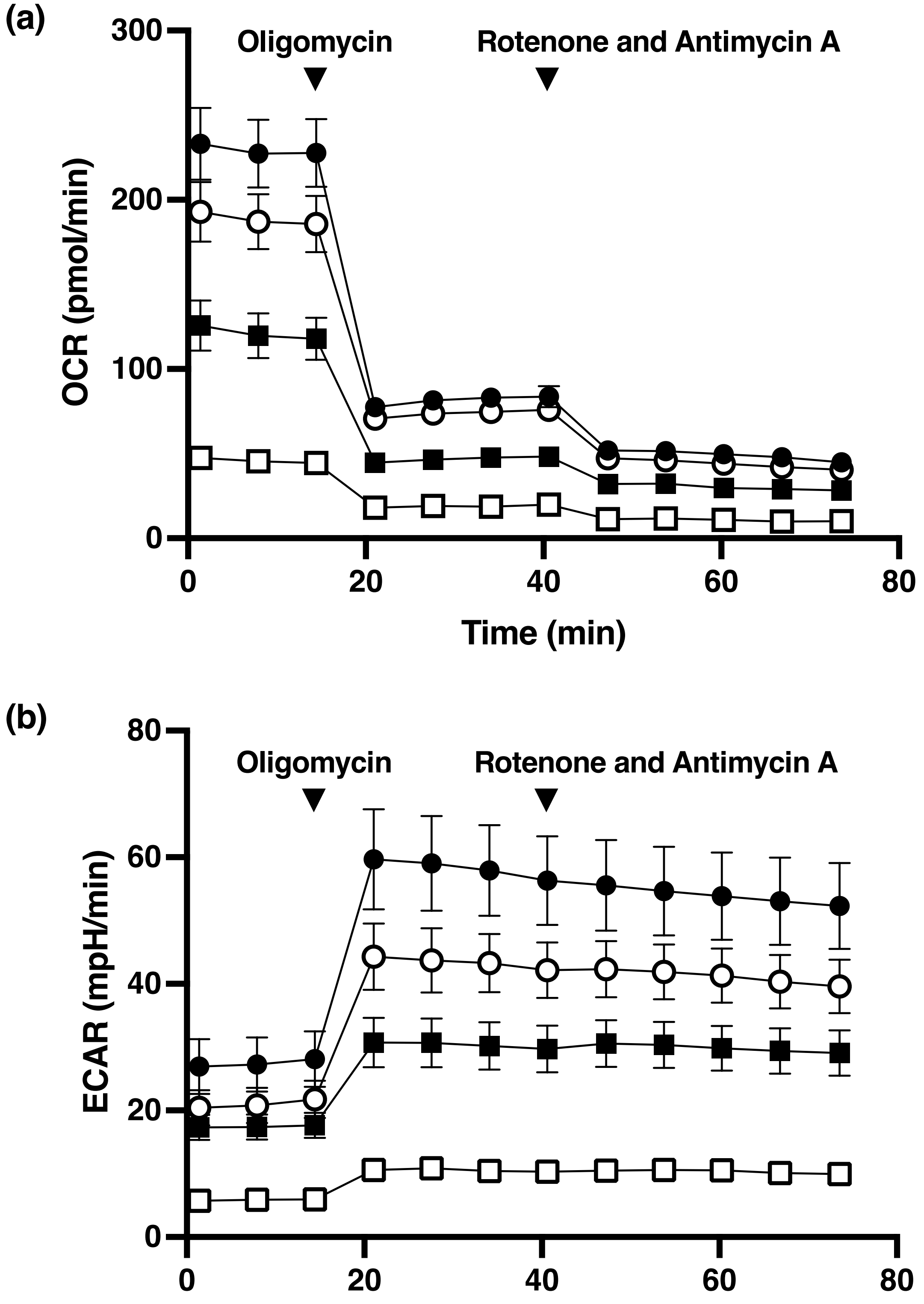

Supplementary Figure 1. Cellular respiration property of 5-FU-resistant HCT116RF10 and parental HCT116 cells to 5-FU under high- and low-glucose culture conditions. The OCR and ECAR were determined using an Agilent Seahorse XFe24 analyzer. (A) OCR. (B) ECAR. The OCR (pmol/min) and ECAR (mpH/min) represent the average of three independent experiments, with error bars showing ±SE. Black circle, HCT116 cells under high glucose; white circle, HCT116 cells under low glucose; black square, HCT116RF10 cells under high glucose; white square, HCT116RF10 cells under low glucose. Oligomycin (final concentration: 1 μM); Rotenone and antimycin A (final concentration: 0.5 μM each).
