# Supplementary material for: Relationship between anticancer sensitivities and cellular respiration properties in 5‐fluorouracil‐resistant HCT116 human colorectal cancer cells
Source: FEBS Open Bio. 2023 Apr 19;13(6):1125–33. doi: 10.1002/2211-5463.13611 (PMC10240340; doi:10.1002/2211-5463.13611)
Supplement: Supplementary file 2 — Table S1. Gene mutation status of glucose metabolism‐related genes in the parental HCT116 cells and 5‐FU‐resistant HCT116RF10 cells. [file FEB4-13-1125-s001.pdf]

Supplementary Table 1. Gene mutation status of glucose metabolism-related genes in the parental HCT116 cells and 5-FU-resistant HCT116R^{F10} cells.

Gene symbol	HCT116	HCT116R^{F10}	Effect
	mt(c.168+22_168+23delTT)het	*wt*	IV
HK1	*wt*	mt(c.168+21_168+23delTTT)het	IV
	wt	mt(c.168+20094T>G)het	IV
HK2	*wt*	mt(c.1238A>G)het	MV (Asp413Gly)
HK3	*wt*	mt(c.2377C>A)het	MV (Leu793Ile)
PFKP	*wt*	mt(c.474C>T)het	SV
ALDOB	mt(c.379+92T>C)het	*wt*	IV
GAPDHS	mt(c.449+247delT)het	*wt*	IV
	mt(c.742-24A>G)het	*wt*	IV
PGAM1	***wt***	**mt(c.-1408_-1402delAAAAAAA)hom**	UGV
	mt(c.139+1515A>T)het	*wt*	IV
ENO3	*wt*	mt(c.85+77T>C)het	IV
ENO4	*wt*	mt(c.936+43_936+47delA5)het	IV
PKM	***wt***	**mt(c.92+2516_92+2519delAAAA)hom**	IV
LDHA	wt	mt(c.922-15delT)het	IV
LDHB	**mt(c.596-9_596-7delTTT)hom**	***wt***	SRV&IV
	wt	mt(c.596-10_596-7delTTTT)het	SRV&IV
	wt	mt(c.596-10_596-8delTTT)het	SRV&IV

	wt	mt(c.421+107A>G)het	IV
LDHAL6B	mt(c.*61C>T)het	*wt*	3’UV
IDH1	mt(c.415-19C>T)het	*wt*	IV
OGDH	mt(c.-27-13_-27-7delTTTTTTT)het	*wt*	SRV&IV
	wt	mt(c.-27-14_-27-7delTTTTTTTT)het	SRV&IV
PDHB	mt(c.304-102_304-101insTAA)het	*wt*	IV
	wt	mt(c.362A>C)het	MV (Asp121Ala)
NDUFS1	mt(c.196-20_196-13delTTTTTTTT)het	*wt*	IV
	wt	mt(c.196-20_196-11delTTTTTTTTTT)het	IV
NDUFS2	mt(c.*86_*107dupGTGTGTGTGTGTGTGTGTGTGT)het	*wt*	3’UV
	wt	mt(c.*80_*107dupGTGTGTGTGTGTGTGTGTGTGTGTGTGT)het	3’UV
NDUFS4	mt(c.98+12952C>A)het	*wt*	IV
	wt	mt(c.*38A>C)het	3’UV
NDUFS5	**mt(c.216+69delT)hom**	***wt***	IV
	wt	mt(c.216+68_216+69delTT)het	IV
NDUFA5	*wt*	mt(c.265-101dupA)het	IV
NDUFAF4	mt(c.241-11dupT)het	*wt*	IV
NDUFAF6	*wt*	mt(c.420+15delA)het	IV

NDUFB2	mt(c.-990_-987delTTTT)het	*wt*	UGV
	wt	mt(c.-990_-986delTTTTT)het	UGV
	wt	mt(c.244-85delT)het	IV
NDUFB2-BRAF	*wt*	mt(n.140726482C>A)het	IR
NDUFB3	**mt(c.*154_*155delAA)hom**	***wt***	DGV
SDHAF3	mt(c.174+18612dupT)het	*wt*	IV
SDHAP1	mt(n.2325+22T>C)het	*wt*	IV
	mt(n.2156C>T)het	*wt*	NTEV
	mt(n.2090-6G>C)het	*wt*	SRV&IV
	mt(n.2089+109T>A)het	*wt*	IV
	wt	mt(n.1609A>G)het	NTEV
	wt	mt(n.1191+79C>A)het	IV
	wt	mt(n.1191+74A>G)het	IV
SDHAP2	mt(n.214-27C>A)het	*wt*	IV
	mt(n.1615-171G>A)het	*wt*	IV
	mt(n.1840G>A)het	*wt*	NTEV
	mt(n.1843G>A)het	*wt*	NTEV
	mt(n.1857+88delT)het	*wt*	IV
	mt(n.1857+109T>A)het	*wt*	IV

mt(n.1857+128dupT)het	*wt*	IV
mt(n.1857+132T>C)het	*wt*	IV
mt(n.1971+84C>T)het	*wt*	IV
mt(n.1971+1880T>A)het	*wt*	IV
mt(n.1971+1898A>T)het	*wt*	IV
mt(n.1971+1974A>G)het	*wt*	IV
wt	mt(n.519A>C)het	SRV&NTEV
wt	mt(n.779A>G)het	NTEV
wt	mt(n.852T>G)het	NTEV
wt	mt(n.1323-49_1323-47delTGG)het	IV
wt	mt(n.1323-35T>C)het	IV
wt	mt(n.1422G>A)het	NTEV
wt	mt(n.1429C>T)het	NTEV
wt	mt(n.1433G>A)het	NTEV
wt	mt(n.1449_1450insTT)het	NTEV
wt	mt(n.1475C>A)het	NTEV
wt	mt(n.1482G>A)het	NTEV
wt	mt(n.1492+69dupT)het	IV
wt	mt(n.1971+2012T>C)het	IV
wt	mt(n.1971+2029A>G)het	IV

	wt	mt(n.1971+2047A>C)het	IV
	wt	mt(n.1971+2123T>C)het	IV
SDHB	mt(c.424-53A>G)het	*Wt*	IV
SDHC	mt(c.77+43delT)het	*Wt*	IV
	**mt(n.210-6delT)hom**	***wt***	SRV&IV
	mt(c.405+103_405+104delTT)het	*wt*	IV
	mt(c.406-15_406-14delTT)het	*wt*	IV
	wt	**mt(c.77+43delT)hom**	VI
	wt	mt(n.210-7_210-6delTT)het	SRV&IV
	wt	mt(n.210-6delT)het	SRV&IV
	wt	**mt(c.405+103_405+104delTT)hom**	IV
UQCRH	*wt*	mt(c.244-103A>G)het	IV
COX7A2	**mt(c.205-11_205-9delTTT)hom**	***wt***	IV
	wt	mt(c.205-12_205-9delTTTT)het	IV
	wt	mt(c.205-12_205-10delTTT)het	IV
COX7B2	*wt*	mt(c.-49-167A>G)het	IV
COX15	**mt(c.272+87_272+93delTTTTTTT)hom**	***wt***	IV
	wt	**mt(c.272+88_272+93delTTTTTT)hom**	IV
COX18	mt(c.607+69A>C)het	*wt*	IV
	mt(c.607+68A>C)het	*wt*	IV

	mt(c.443+1353_443+1358delCACACA)het	*wt*	IV
	wt	mt(c.607+66A>C)het	IV
	wt	mt(c.443+1401T>A)het	IV
	wt	mt(c.443+1353_443+1360delCACACACA)het	IV
ATP5I	mt(c.191-84_191-80delAAAAA)het	*wt*	IV
ATP5J2-	mt(c.122-7391_122-7388delTTTT)het	*wt*	IV
PTCD1	mt(c.121+10013A>G)het	*wt*	IV
	wt	**mt(c.122-7390_122-7388delTTT)hom**	IV
ATP6AP1L	mt(c.132-77T>C)het	*wt*	IV
ATP6V1A	mt(c.1761+63delT)het	*wt*	IV
	mt(c.1762-69_1762-68delAA)het	*wt*	IV
	wt	**mt(c.1762-69_1762-68delAA)hom**	IV
ATP6V1B1	*wt*	mt(c.1155delC)het	FV
ATP6V1C1	**mt(c.133-43delT)hom**	***wt***	IV
	mt(c.642-8_642-7delTT)het	*wt*	SRV&IV
	mt(c.642-7delT)het	*wt*	SRV&IV
	mt(c.894dupA)het	*wt*	FV
	wt	mt(c.-39-52_-39-51delTT)het	IV
	wt	mt(c.133-44_133-43delTT)het	IV
	wt	mt(c.133-43delT)het	IV

	wt	**mt(c.642-8_642-7delTT)hom**	SRV&IV
ATP6V0A4	**mt(c.640-21delT)hom**	***wt***	IV
	wt	mt(c.640-21delT)het	IV
	wt	mt(c.640-29T>C)het	IV
ATP6V0E1	mt(c.105-4_105-3delTT)het	*wt*	SRV
	wt	mt(c.105-5_105-3delTTT)het	SRV
ATP6V0E2	*wt*	**mt(c.368+94_368+95insGTGGTTAGAGTTCTTGTTGG GATTCAGGCATCTATTTATTTCATGAAAAGAAAAG GTGGGGAGGGGAC)hom**	IV
ATP8A1	*wt*	mt(c.2897-4A>G)het	SRV&IV
	wt	**mt(c.1413+122_1413+125dupTTTA)hom**	IV
	wt	mt(c.450+98T>G)het	IV

Note. *wt*, wild type; mt, mutation-type; hom, homozygous; het, heterozygous; IV, intron variant; MV, missense variant; SV, synonymous variant; UGV, upstream gene variant; SRV, splice region variant; 3’UV, 3’-UTR variant; FV, frameshift variant; DGV, downstream gene variant; NTEV, noncoding transcript exon variant; IR, intergenic region.
